# Ferric Carboxymaltose in the Management of Iron Deficiency Anemia in Pregnancy: A Subgroup Analysis of a Multicenter Real-World Study Involving 1191 Pregnant Women

**DOI:** 10.1155/2022/5759740

**Published:** 2022-11-28

**Authors:** Prakash Trivedi, S. Chitra, Suma Natarajan, Vandana Amin, Shilpi Sud, Priti Vyas, Meenakshi Singla, Ajinkya Rodge, Onkar C. Swami

**Affiliations:** ^1^Dr. Trivedi's Total Women Health Care Hospital, Mumbai, Maharashtra, India; ^2^Lalitha Nursing Home, Tiruchirappalli, Tamil Nadu, India; ^3^Ganga Medical Centre & Hospitals, Coimbatore, Tamil Nadu, India; ^4^Lady Care Women's Hospital & Child Care, Ahmedabad, Gujarat, India; ^5^Safal Hospital, Nagpur, Maharashtra, India; ^6^Sangita Maternity Surgical and Diagnostic Centre, Mumbai, Maharashtra, India; ^7^Dr. Meenakshi Maternity and Child Clinic, Rohini, Delhi, India; ^8^Emcure Pharmaceuticals Ltd, Pune, Maharashtra, India

## Abstract

**Background:**

Real-world evidence of the efficacy and safety of ferric carboxymaltose (FCM) infusion in Indian pregnant women with iron deficiency anemia (IDA) is lacking.

**Objective:**

To assess the efficacy and safety of intravenous (IV) FCM in Indian pregnant women with IDA in 4 weeks in a real-life scenario.

**Methods:**

This is a subgroup analysis of our previously conducted retrospective, multicenter, observational, real-world PROMISE study. Data on demographic and hematological parameters, patient-reported adverse events, and physicians' clinical impressions of efficacy and safety were analysed at 4 ± 1 week.

**Results:**

This subgroup analysis included 1191 pregnant women in whom IV FCM resulted in a significant increase in hemoglobin (Hb) by 2.8 g/dL and serum ferritin by 30.03 *μ*g/L at 4 weeks (*P* < 0.001 for both). In 103 pregnant women with severe IDA, there was a significant increase in Hb by 3.6 g/dL (*P* < 0.001), and serum ferritin by 16.96 *μ*g/L (*P*=0.12). In 978 pregnant women with moderate IDA, significant improvement in Hb by 2.74 g/dL and serum ferritin by 33 *μ*g/L (*P* < 0.001 for both) was noted. Similarly, there was a significant increase in red blood cell count, hematocrit, mean corpuscular volume, and mean corpuscular hemoglobin (*P* < 0.001 for all). In pregnant women with mild IDA (*n* = 26), Hb increased significantly by 1.99 g/dL (*P* < 0.001). Adverse effects were reported in 8.6% of pregnant women. No new safety signals or serious adverse effects were observed. Based on physicians' global assessment, good to very good efficacy and safety of IV FCM was noted in 99.2% and 98.6% of pregnant women, respectively.

**Conclusions:**

IV FCM rapidly corrected anemia in a short period of 4 weeks with favorable safety in the second and third trimester of pregnancy with all severities of IDA (severe, moderate, and mild). The physicians' favorable global assessment of FCM's efficacy and safety in pregnant women with IDA supports its use in daily clinical practice. This trial is registered with CTRI/2021/12/039065.

## 1. Introduction

Anemia is a serious global health concern that commonly affects pregnant women [1]. The World Health Organization (WHO) reported globally that 40% of pregnant women have anemia [1]. According to the World Bank data, 48% of pregnant women in South Asia and 50% of pregnant women in India had anemia in 2019 [2]. The National Family Health Survey (NFHS-5; 2019-2020) data reported that 52.2% of Indian pregnant women have anemia [3]. Two recent studies from India reported anemia in 81.8% (91% had moderately severe anemia) [4] and 90% (60.5% had moderately severe anemia) [5] of pregnant women. Despite the availability of treatments and guidelines [6, 7], minimal improvement is witnessed in the anemia status of pregnant women in India.

Iron deficiency anemia (IDA) accounts for 75% of anemia cases in pregnancy [8]. The iron deficiency is more severe in pregnancy because of the increased iron demand [4, 7]. Further, maternal iron demand increases in the second and third trimester of pregnancy as the majority of iron transfer to the fetus occurs during this period [7]. Maternal anemia also results in adverse maternal and neonatal outcomes, which are proportional to the increasing severity of anemia [9]. Anemia in pregnancy accounts for 20% of maternal and 18% of perinatal mortality in South Asian countries including India [10]. Therefore, prompt correction of anemia in pregnancy is needed, and this limits the use of oral iron preparations, especially in moderate to severe anemia [11].

Ferric carboxymaltose (FCM) is a third-generation parenteral iron formulation used for correcting IDA. There are many Indian [12–20] and international [21–24] clinical studies supporting the efficacy and safety of FCM in treating IDA in pregnancy. However, there is limited real-world evidence (RWE) [25–29] reporting the efficacy and safety of FCM in pregnancy and particularly in India. Many of these Indian studies are of small sample size and reported from a single center. RWEs are important because they substantiate the clinical trial evidence in real-world scenarios [30]. The previously reported PROMISE study is a retrospective, observational, real-world study of 1800 patients with IDA that assessed the efficacy and safety of intravenous (IV) FCM in adolescents and adults with IDA [31]. Herewith, we represent the efficacy and safety of IV FCM for correcting anemia in a subgroup of 1191 pregnant Indian women with IDA.

## 2. Materials and Methods

### 2.1. Study Design, Subject, and Treatment Characteristics

This is a subgroup analysis of a multi-center, retrospective, observational, data collection study (PROMISE) across 269 centers in India in a real-world scenario [31]. Pregnant women with IDA (hemoglobin (Hb) level between 4 and <12 g/dL), who provided informed consent for future use of their medical records for research and received IV FCM were included in the PROMISE study. The following subjects were excluded from the PROMISE: anemia other than IDA; severe iron deficiency with Hb < 4 g/dL; first trimester pregnancy; known hypersensitivity to FCM or its excipients, or other IV iron products; malignancy; iron overload conditions (e.g. hemochromatosis/hemosiderosis); participant considered unsuitable by the investigator.

The subgroup analysis was performed with data of pregnant women who received Injection FCM 500/1000 mg infusion (Orofer FCM, Emcure Pharmaceuticals Ltd., Pune, India; not exceeding 1000 mg iron per infusion) between January 1, 2021 and December 31, 2021 in real-life clinical practice. The cumulative FCM dose for iron repletion was determined based on the subject's body weight and Hb level and is detailed in Table 1.

### 2.2. Outcome Measures and Statistical Analyses

Available data on hematological parameters (Hb, serum ferritin, and so on) at baseline and/or at minimum of 4 ± 1 week (henceforth reported as 4 weeks) were anonymously captured from the subjects' medical records. Demographic and hematological parameters were analysed using descriptive statistical methods. Data were synthesized for the entire study population and by the severity of anemia. Quantitative data were described as the mean ± standard deviation (SD). Categorical data were represented as frequencies and percentages. A paired *T*-test was carried out to compare the hematological parameters at baseline and 4 weeks after FCM infusion.

Hb values ≥11 g/dL were considered normal as per WHO Hb cut-off values for anemia in pregnancy [32]. Anemia was categorized as mild, moderate, and severe based on the WHO's Hb cut-off values [32]: severe anemia (Hb < 7 g/dL); moderate anemia (Hb 7 to 9.9 g/dL); and mild anemia (Hb 10–10.9 g/dL).

Efficacy was assessed based on the hematological improvement seen and safety was assessed based on the occurrence of adverse events throughout the study duration. Physicians' global assessment of the efficacy and safety of FCM in their subjects were graded as very good, good, average, or poor.

## 3. Results

### 3.1. Baseline Characteristics

Data of 1191 pregnant women was included in this subanalysis; the mean age was 30.33 years (range 19 to 48 years); with a mean Hb of 8.03 g/dL and a mean serum ferritin at 40.06 *μ*g/L. Other demographic and hematological parameters at baseline are shown in (Table 2). The mean weeks of gestation were 28.3 weeks. The mean cumulative FCM dose was 1027 mg, and the average FCM infusion time was 18.10 minutes. Hypertension, diabetes, hookworm infestation, and kidney disease were seen in a small percentage of the study population (Table 2).

### 3.2. Efficacy Outcomes

There was a significant increase in Hb by 2.8 g/dL and serum ferritin by 30 *μ*g/L at 4 weeks (*P* < 0.001 for both). Similarly, there was a significant increase in red blood cell (RBC) count, hematocrit, mean corpuscular volume (MCV), and mean corpuscular hemoglobin (MCH) (*P* < 0.001 for all). The mean corpuscular hemoglobin concentration (MCHC) increased at 4 weeks (*P*=0.135) (Table 3).

In pregnant women with severe IDA (*n* = 103), a significant increase in Hb by 3.6 g/dL at 4 weeks was noted (*P* < 0.001). The serum ferritin increased by 16.96 *μ*g/L (*P*=0.12). A significant increase in RBC count and MCH at 4 weeks (*P* < 0.001 for both) was also noted. MCHC MCV, and hematocrit improved at 4 weeks as compared to baseline (MCHC: *P*=0.47, MCV: *P*=0.086, and hematocrit: *P*=0.735) (Table 4).

A significant rise in Hb by 2.74 g/dL and serum ferritin by 33 *μ*g/L was noted in pregnant women with moderate IDA (*n* = 978), (*P* < 0.001 for both) (Table 4); similarly, there was a significant increase in RBC count, hematocrit, MCV, and MCH (*P* < 0.001 for all). The improvement in MCHC at 4 weeks was noted (*P*=0.179).

In pregnant women with mild IDA (*n* = 26), significant rise in Hb of 1.99 g/dL (*P* < 0.001) was noted. The other hematological parameters improved at 4 weeks (Table 4): serum ferritin (*P*=0.318), RBC count (*P*=0.5), hematocrit (*P*=0.214), MCH (*P*=0.066), and MCHC (*P*=0.991).

### 3.3. Safety

Adverse effects (AEs) were observed in 8.6% of pregnant women (103/1191). The commonly reported AEs were: nausea (5%), headache (3.7%), constipation (0.2%), and allergic reaction (0.2%). No serious adverse events (SAEs) were reported in any of the subjects.

### 3.4. Physician Reported Outcomes

**Figure 1:**
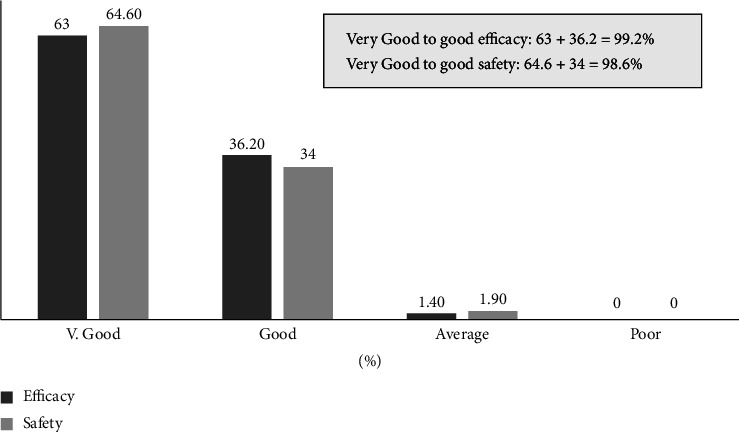
Physicians' assessment on efficacy and safety of ferric carboxymaltose in pregnant women.

Good to very good efficacy of FCM was noted in 99.2% of pregnant women. Poor response was noted in none of pregnant women (Figure 1). Good to very good safety was reported in 98.6% of pregnant women. None of the pregnant women reported poor tolerability (Figure 1).

## 4. Discussion

The NFHS-5 (2019-2020) data showed that anemia is prevalent in more than half (52.2%) of all Indian pregnant women [3]. IDA accounts for 75% of anemia cases in pregnancy [8]. Thus, anemia is a significant health concern in pregnancy in India. Further, maternal iron demand increases in the second and third trimester of pregnancy as the majority of iron transfer to the fetus occurs during this period [7]. Thus, the average daily iron requirement of a pregnant woman increases from 0.8 mg/day in the first trimester to 7.5 mg/day in the third trimester [33]. Thus, the iron deficiency becomes more severe in pregnancy because of the increased iron demand [4, 7].

The present subgroup analysis showed that pregnant women (*n* = 1191; 62.2%) comprised a significant proportion of IV FCM-treated PROMISE study subjects with IDA (*N* = 1800). Additionally, moderate (59.3% to 91%), moderate-to-severe (60.5%), and severe anemia (8.8% to 13.1%) are common in Indian pregnant women [4, 5, 20, 33]. In line with this previously reported data from India, our study also showed that 82.1% of the included pregnant women had moderate anemia and 8.6% had severe anemia. Therefore, it can be inferred that moderate to severe anemia continues to be a significant health concern during the second and third trimester in pregnant women in India.

If not corrected promptly, maternal anemia significantly impacts perinatal, neonatal and maternal outcomes, conferring a significantly higher risk of perinatal mortality, preterm birth, low birth weight, neonatal, and maternal morbidity and mortality compared to pregnant women without anemia [10, 34]. Thus, timely diagnosis and management of anemia in pregnancy are crucial for preventing adverse outcomes [11]. However, a major problem noted in India is that women often present for their first antenatal visit in the second or third trimester [5]. Thus, anemia is diagnosed late, thereby increasing the risk of adverse maternal and neonatal outcomes. Hence, there is an urgent need to restore iron stores and correct the anemia quickly to prevent these adverse maternal and neonatal outcomes. This need can be adequately fulfilled with parenteral iron therapy and the parenteral iron used in pregnancy should be fast-acting, must not cross the placenta, and have the properties that allow large doses to be administered safely in the second and third trimester of pregnancy [7, 11]. FCM matches all these properties of ideal parenteral iron, and therefore is a good therapeutic option for rapid correction of anemia during pregnancy [11].

There is abundant clinical evidence supporting the effective and safe use of IV FCM for correcting anemia and replenishing iron stores during pregnancy [12–24]. Also, compared to other parenteral iron preparations, IV FCM reported rapid correction of anemia during pregnancy [12–24]. Further, a large FCM dose (1000 mg) can be administered in a single setting with good efficacy and minimal adverse effects [12, 14–16, 21, 23, 24, 27]. This is important for compliance in resource-limited settings [25].

In this context, a different recently published real-world study involving pregnant women from India reported that IV FCM significantly and rapidly improved Hb levels by 4.23 g/dL in severe IDA at 4 weeks (*P* < 0.001) [25]. The significant increase in Hb was seen as early as day 20 after IV FCM (*P* < 0.001). Also, IV FCM resulted in a significant improvement in Hb in pregnant women who received FCM after 34 weeks of gestation (*P*=0.002) [25]. Similarly, the present study highlighted that within a short span of 4 weeks, IV FCM significantly increased Hb by 3.6 g/dL in pregnant women with severe anemia and a significant rise in Hb by 2.74 g/dL and serum ferritin by 33 *μ*g/L in pregnant women with moderate IDA (*P* < 0.001 for all).

Despite a good clinical profile in pregnancy, there are very limited RWE studies [25–29] substantiating the efficacy and safety of FCM for managing IDA during pregnancy. The REGAIN retrospective study (*N* = 1001) showed that the there was a direct relationship between the FCM dose and increase in Hb levels [26]. The majority of study participants (70.3%) received 1000 mg of FCM, and this resulted in an increase of Hb by ≥2 g/dL in 39.2% of pregnant women on this dose [26]. The present subgroup analysis of the PROMISE study showed that large doses of FCM (1000 mg) administered as a single infusion resulted in rapid improvement in Hb, iron stores, and other hematological parameters within 4 weeks of FCM infusion.

Compared to other parenteral iron preparations, FCM has an excellent safety profile in pregnancy [25], with no [14] or minimal mild adverse effects reported by fewer patients [12, 15, 16, 23, 24]. The most common adverse effects reported, usually after ≥2 FCM doses are headache, mild local reaction, nausea, dizziness, abdominal pain, constipation, and fever and chills [12, 15, 16, 23, 24]. The present subgroup analysis also reported minimal adverse effects in 8.6% of study population which is in line with the published data. No new safety signals were noted, and no SAEs were reported in this study. Thus, the present analysis demonstrated that FCM is a safe treatment option for correcting IDA in pregnant women in real-life setting in resource-limited settings.

The study is limited by its retrospective design, missing data, and the fact that few subjects received two FCM 500 mg infusions instead of a single 1000 mg infusion. However, to the best of our knowledge, this is the largest real-world study (*N* = 1191) in pregnant women with IDA demonstrating the efficacy and safety of FCM in real-life management scenarios. Also, though health-related quality of life improvement after FCM administration is often reported [23, 24], literature on physicians' assessment of the efficacy and safety of FCM is lacking. The present study highlights the excellent efficacy and safety of FCM based on physicians' reported global assessment. Good to very good efficacy and safety were noted in 99.2% and 98.6% of pregnant women, respectively.

## 5. Conclusions

The present large real-world evidence supports clinical place of IV FCM in management of IDA in pregnant women. Rapid and significant improvement in hematological parameters with favorable tolerability was noted in a large cohort of 1191 pregnant women with IDA. In a resource-limited setting, single-dose administration, a rapid improvement of hematological parameters with favorable tolerability makes FCM as the best-suited option for the management of moderate-to-severe IDA during pregnancy [35].

## Figures and Tables

**Table 1:** Cumulative FCM dose for iron repletion.

Body weights	Hb < 10 g/dL	Hb 10–14 g/dL
	Maximum FCM dose allowed	
<35 kg	500 mg	500 mg
35 kg to <70 kg	1500 mg	1000 mg
≥70 kg	2000 mg	1500 mg

*Note.* Maximum tolerated single dose: 1000 mg of iron (20 ml) per day. Do not administer 1000 mg of iron (20 ml) more than once a week. FCM, ferric carboxymaltose; g/dL, grams per deciliter; Hb, hemoglobin; mg, milligram; kg, kilograms.

**Table 2:** Subject characteristics at baseline.

	*N*	Mean ± SD	Median (IQR)	Range (min–max)
Age (years)	1155	30.33 ± 4.77	30 (27, 32)	19 to 48
Weight (kilogram)	1086	57.35 ± 9.37	56 (50, 64)	30 to 98
Weeks of gestation	775	28.34 ± 4.21	28 (24, 32)	14 to 39
Hemoglobin (g/dL)	1133	8.03 ± 0.9	8 (7.5, 8.7)	5.2 to 11.04
Serum ferritin (*μ*g/L)	286	40.06 ± 45.13	29 (7.79, 58)	0.1 to 238
RBC count (mn/mm3)	296	3.87 ± 0.75	3.9 (3.4, 4.2)	2 to 6.8
Hematocrit (%)	270	32.26 ± 5.77	31.3 (29, 36.05)	20 to 46
MCV (fL)	280	68.99 ± 10.98	69.2 (62.13, 75)	11.3 to 102.1
MCH (pg)	278	24.22 ± 6.23	22.6 (20.21, 29)	2.8 to 38.8
MCHC (g/dL)	273	29.73 ± 3.01	29.8 (28.4, 31.35)	14 to 39

*Comorbidities at baseline*				
		*N* (%)		
Hypertension		28 (2.4%)		
Diabetes		15 (1.3%)		
Hookworm infestation		2 (0.2%)		
Kidney disease		4 (0.3%)		

%-percentage; *μ*g/L-micrograms per liter; fL-femtoliters; g/dL-grams per deciliter; IQR-interquartile range; MCH-mean corpuscular hemoglobin; MCHC-mean corpuscular hemoglobin concentration; MCV-mean corpuscular volume; Min-Max-minimum-maximum; mn/mm^3^-million per millimeter cube; *N*-number of participants; pg-picograms; RBC-red blood cell; SD-standard deviation. *Note*. 4 weeks is 4 ± 1 week.

**Table 3:** Comparing hematological parameters before and after administration of ferric carboxymaltose in pregnant women.

Parameters	*N*	At baseline (mean ± SD)	At 4 weeks (mean ± SD)	Mean improvement ± SD
Hemoglobin (g/dL)	1107	8.03 ± 0.9	10.83 ± 1.07	2.8 ± 1.02^*∗*^
Ferritin (*μ*g/L)	251	38.79 ± 46.91	68.81 ± 68.58	30.03 ± 53.77^*∗*^
RBC (mn/mm^3^)	214	4.09 ± 0.7	4.66 ± 0.91	0.57 ± 1.09^*∗*^
Hematocrit (%)	204	33.31 ± 5.62	35.21 ± 8.16	1.9 ± 6.35^*∗*^
MCV (fL)	249	69.47 ± 10.18	75.99 ± 17.69	6.52 ± 20.79^*∗*^
MCH (pg)	247	24.37 ± 6.36	27.83 ± 6.13	3.46 ± 7.34^*∗*^
MCHC (g/dL)	244	29.56 ± 2.99	30.95 ± 15.09	1.38 ± 14.41^NS^

^*∗*^ *P* value < 0.001, statistically significant difference; NS-*P* value >0.05, non-significant difference. %-percentage; *μ*g/L-micrograms per liter; fL-femtoliters; g/dL-grams per deciliter; MCH-mean corpuscular hemoglobin; MCHC-mean corpuscular hemoglobin concentration; MCV-mean corpuscular volume; mn/mm3-million per millimeter cube; *N*-number of participants; pg-picograms; RBC-red blood cell; SD-standard deviation. *Note*: 4 weeks is 4 ± 1 week.

**Table 4:** Comparing hematological parameters before and after administration of ferric carboxymaltose by the severity of anemia

Severity of anemia	Parameters	*N*	At baseline (mean ± SD)	At 4 weeks (mean ± SD)	Mean improvement ± SD
Severe	Hemoglobin (g/dL)	103	6.49 ± 0.41	10.09 ± 1.11	3.6 ± 1.08^*∗*^
	Ferritin (*μ*g/L)	46	51.88 ± 58.3	68.84 ± 39.47	16.96 ± 72.52^NS^
	RBC (mn/mm^3^)	38	3.53 ± 0.59	4.56 ± 0.89	1.03 ± 1.22^*∗*^
	Hematocrit (%)	37	32.82 ± 7.74	32.22 ± 14.28	0.6 ± 10.7^NS^
	MCV (fL)	46	75.82 ± 11.35	68.61 ± 25.62	7.2 ± 27.87^NS^
	MCH (pg)	45	25.75 ± 6.15	28.03 ± 5.59	2.28 ± 6.16^#^
	MCHC (g/dL)	44	28.65 ± 3.52	29.67 ± 10.52	1.03 ± 9.34^NS^

Moderate	Hemoglobin (g/dL)	978	8.14 ± 0.72	10.87 ± 1.03	2.74 ± 0.98^*∗*^
	Ferritin (*μ*g/L)	203	36.13 ± 43.7	69.12 ± 73.88	33 ± 48.54^*∗*^
	RBC (mn/mm^3^)	174	4.21 ± 0.67	4.68 ± 0.92	0.47 ± 1.04^*∗*^
	Hematocrit (%)	165	33.43 ± 5.09	35.89 ± 5.92	2.46 ± 4.79^*∗*^
	MCV (fL)	202	68.07 ± 9.35	77.64 ± 14.96	9.58 ± 17.49^*∗*^
	MCH (pg)	200	24.04 ± 6.38	27.73 ± 6.23	3.68 ± 7.6^*∗*^
	MCHC (g/dL)	198	29.75 ± 2.83	31.22 ± 16.01	1.48 ± 15.39^NS^

Mild	Hemoglobin (g/dL)	26	10.18 ± 0.25	12.17 ± 0.77	1.99 ± 0.8^*∗*^
	Ferritin (*μ*g/L)	2	8 ± 9.9	37.1 ± 32.39	29.1 ± 22.49^NS^
	RBC (mn/mm^3^)	2	3.93 ± 0.1	4.13 ± 0.18	0.2 ± 0.28^NS^
	Hematocrit (%)	2	32.25 ± 1.34	34.25 ± 2.33	2 ± 0.99^NS^
	MCV (fL)	1	61.4 ± 0	80.6 ± 0	—
	MCH (pg)	2	26.15 ± 8.27	33.4 ± 9.33	7.25 ± 1.06^NS^
	MCHC (g/dL)	2	31.6 ± 2.55	31.65 ± 2.33	0.05 ± 4.88^NS^

^*∗*^ *P* value <0.001, statistically significant difference; NS-*P* value >0.05, nonsignificant difference. %-percentage; *μ*g/L-micrograms per liter; fL-femtoliters; g/dL-grams per deciliter; MCH-mean corpuscular hemoglobin; MCHC-mean corpuscular hemoglobin concentration; MCV-mean corpuscular volume; mn/mm3-million per millimeter cube; *N*-number of participants; pg-pictograms; RBC-red blood cell; SD-standard deviation. *Note*: 4 weeks is 4 ± 1 week.

## Data Availability

It will be provided on request.
